# Association between predicted fat mass, predicted lean mass, predicted percent fat and type 2 diabetes mellitus in Japanese adults: a retrospective study

**DOI:** 10.1186/s12902-024-01579-4

**Published:** 2024-04-17

**Authors:** Jiaming Tang, Xiaohua Cai, Aijie Liu, Nannan Yu, Shilei Wang

**Affiliations:** 1 Department of Anesthesiology, Affiliated Hospital of Qingdao University, Qingdao, Shandong China; 2 Department of Pharmacy, Qingdao Hospital, University of Health and Rehabilitation Sciences (Qingdao Municipal Hospital), Qingdao, Shandong China; 3 Department of Geriatrics, Qingdao Hospital, University of Health and Rehabilitation Sciences (Qingdao Municipal Hospital), Qingdao, Shandong China

**Keywords:** Type 2 diabetes mellitus, Predicted fat mass, Predicted lean mass, Predicted percent fat

## Abstract

**Background:**

Type 2 diabetes mellitus (T2DM) is known to have obesity as a risk factor. Body mass index cannot distinguish between lean mass and fat mass. We aimed to examine the association between predicted fat mass, predicted lean mass, predicted percent fat and risk of T2DM in Japanese adults. We also explored whether these three new parameters could predict T2DM better than other obesity markers.

**Methods:**

This present study is a secondary data analysis. The study enrolled 20,944 Japanese individuals who participated in the NAGALA medical assessment program between 2004 and 2015. 15,453 participants who are eligible and have complete information were included to our analysis. Through the use of Kaplan-Meier curve, restricted cubic spline and univariate and multivariate Cox regression analysis, the relationship between predicted fat mass, predicted lean mass, predicted percent fat and T2DM risk was examined. The area under the curve method was used to assess the differences between these markers of obesity.

**Results:**

A total of 373 cases of T2DM occurred over a median time of 5.4 years. In the male group, we found a U-shaped connection between predicted fat mass, predicted lean mass, and T2DM onset (*p* value, non-linearity < 0.05). A linear relationship was found between predicted percent fat and T2DM onset. The linear relationship was also found in the female group for predicted fat mass, and predicted percent fat. And for women, predicted lean mass was not an independent predictor. The area under the curve (AUC) for predicted fat mass, predicted lean mass, predicted percent fat in men was 0.673 (95%CI: 0.639 ~ 0.707), 0.598 (95%CI: 0.561 ~ 0.635), 0.715 (95%CI: 0.684 ~ 0.745), respectively. In males, WHtR was the strongest predictor (AUC 0.7151, 95%CI: 0.684 ~ 0.746), followed by predicted percent fat (AUC 0.7150, 95%CI: 0.684 ~ 0.745). In the females, WHtR was also the strongest predictor (AUC 0.758, 95%CI: 0.703 ~ 0.813), followed by body mass index (AUC 0.757, 95%CI: 0.704 ~ 0.811) and predicted percent fat (AUC 0.742, 95%CI: 0.687 ~ 0.798).

**Conclusion:**

Predicted fat mass, predicted lean mass, predicted percent fat were strongly connected with an increased risk for developing T2DM in Japanese, particularly in males. WHtR and predicted percent fat had a slightly better discrimination than other common obesity indicators in males. In the females, predicted fat mass and predicted percent fat were associated with T2DM risk, WHtR and body mass index had the slightly higher predictive power.

**Supplementary Information:**

The online version contains supplementary material available at 10.1186/s12902-024-01579-4.

## Introduction

Type 2 diabetes (T2DM) is characterized by insulin resistance and pancreatic beta-cell dysfunction. The prevalence and incidence of T2DM, which accounts for more than 90% of all diabetes cases, is rapidly increasing worldwide. Diabetes prevalence among people aged 20–79 years worldwide is estimated at 536.6 million in 2021, and at 783.2 million in 2045 [1]. As the prevalence increases, the economic cost of T2DM to health systems has increased alarmingly. Therefore, it is vital to identify individuals at high risk of T2DM and provide them with immediate intervention, treatment and management.

T2DM has a complicated etiology that is known to be influenced by a number of risk factors. Obesity is a known and important risk factor for T2DM [2]. The relation of obesity and T2DM is complex. Different obesity indices have been linked to T2DM in numerous studies [3, 4]. An analysis of Mendelian randomization studies found that higher genetically predicted BMI was associated with an increased T2DM risk [5]. However, a number of studies have indicated that BMI is may not be an accurate measure of obesity, especially when it is measured from self-reported weight or height at interview [6, 7]. BMI have been reported to have poor sensitivity and specificity [8], and is mostly used as a screening measure. In many studies, BMI does not take age, sex and bone structure into account, and also does not distinguish between fat mass and lean mass in people with different body composition [7]. Despite having the same BMI, people may have different body compositions. BMI and body fat percentage do not correlate linearly and differ between men and women [8]. Having a lean body mass (mostly skeletal muscles) is protective, whereas having a fat body mass can be detrimental [9, 10]. T2DM has been shown to be associated with worse muscle quality and reduced muscle strength in older persons [11]. Consequently, lean body mass and fat mass may contribute differently to the risk of T2DM. Understanding the link between lean body mass and fat mass in T2DM patients could help our clinical treatment.

Direct assessments of fat mass and lean body mass are not feasible in large-scale investigations because they call for expensive, advanced techniques like dual-energy X-ray absorptiometry (DXA), experienced surveyors, and a lot of time. There are not many studies on the connection between risk of T2DM and directly measured fat mass, lean body mass, and percent fat. It is therefore inappropriate for regular clinical use. Indeed, using large population samples from the National Health and Nutrition Examination Survey (NHANES), a cross-sectional survey based on individual age, race, weight, height, and waist circumference, Lee et al. created validated sex-specific anthropometric prediction equations for predicted fat mass and lean mass [12]. Therefore, we investigated the relationships between predicted FM, predicted LM, predicted PF and risk of T2DM in Japanese individuals using these equations to assess body composition. We further contrasted the relationship between risk of T2DM and predicted FM, predicted LM, predicted PF, BMI, WC, and WHtR.

## Materials and methods

### Data source

The Dryad database (http://www.Datadryad.org/) was utilized for the data. The information was added to the Dryad database by Professor Takuro Okamura and colleagues (Dryad Digital Repository: 10.5061/dryad.8q0p192) [4]. Data package can be downloaded from the database and is free to use by researchers for further analysis. A total of 15,774 patient data sets were collected for use in the study. Each participant supplied written informed consent before to taking part, and this consent was authorized by the Murakami Memorial Hospital ethics committee. As a result, using these data for secondary analysis would not be against the authors’ rights.

### Study population

Patients at Murakami Memorial Hospital who took part in the medical evaluation program between 2004 and 2015 were enrolled in the study. Participants with known liver illness, baseline medication use, T2DM or fasting plasma glucose over 6.1mmol/L, alcohol intake of more than 60 g/day for males and 40 g/day for women, or missing data were not included in this study. HbA1c ≥ 6.5%, fasting plasma glucose (FPG) ≥ 7.0mmol/L, or self-reported clinically diagnosed diabetes were the ADA’s definitions of incident T2DM [13].

### Data collection and measurements

Based on a standardized self-administered questionnaire, the database contained data on each participant’s medical history and lifestyle traits. Age, sex, exercise habits, smoking status, alcohol consumption, fatty liver, weight, body mass index (BMI), waist circumference (WC), total cholesterol (TC), triglyceride (TG), high-density lipoprotein cholesterol (HDL), hemoglobin A1c (HbA1c), fasting plasma glucose (FPG), aspartate aminotransferase (AST), alanine aminotransferase (ALT), g-glutamyl transferase (GGT), systolic blood pressure (SBP), diastolic blood pressure (DBP) levels, new-onset type 2 diabetes mellitus (T2DM), and follow-up times were all factors that were gathered from participants who were enrolled in the study and entered into the database.

### Exposure variables

We calculated predicted FM, LM, PF using prediction equations which were developed and validated by the NHANES database. Previous studies have outlined the derivation of the Eq. [12], as well as basic demographics and anthropometrics, such as age, race, weight, height, and WC. The independent data set’s validation tests revealed no evidence of bias. When stratified by age, BMI, smoking status, race or ethnicity, and sickness status, the equations also performed well. Lastly, there were similar correlations with obesity-related biomarkers between predicted fat mass and DXA measured fat mass. Table S1 (supplemental table S1) displays the anthropometric prediction formulae for each sex.

### Statistical analysis

The data, which were split into continuous variables and categorical variables, were reported using the mean ± standard deviation, the median ± interquartile range (IQR), or numbers (percentages), as applicable. The Student’s t-test, Wilcoxon rank-sum test, and Chi-square test, respectively, were used to do statistical comparisons. Comparisons between the various groups were made using either the one-way analysis of variance or the non-parametric Kruskal-Wallis test. All of the variables were handled as sex-specific tertiles. To examine the impact of these three factors on the likelihood of developing T2DM, Cox regression analysis was used. A hazard ratio (HR) with a 95% confidence interval (95% CI) was used to represent the risk. Using restricted cubic spline analysis, the linear association between FM, LM, PF, and newly-onset T2DM was evaluated. The cumulative occurrences of T2DM across tertiles were represented using the Kaplan-Meier method. In order to evaluate the reliability of the findings, subgroup analyses were conducted. Age, exercise habits, alcohol intake, smoking status, and HBP were divided into subgroups, and these subgroups were then subjected to a stratified linear regression model and likelihood ratio test. The Harrell’s concordance index (C-index) and receiver operating characteristic (ROC) curve analysis were used to gauge and compare the discriminative strength of various parameters.

R (http://www.R-project.org, The R Foundation) and Free Statistics software version 1.7 were used for all statistical studies. All tests were two-sided, and *p*-value < 0.05 was considered statistically significant.

## Results

### General characteristics of the study population

Figure S1 (supplemental figure S1) depicts the flowchart for the patient selection process. In our analysis, we used the remaining 15,453 (8419 men and 7034 women) subjects with complete data, with a mean age of 43.7 ± 8.9 years. 286 men and 87 women were found to have new-onset T2DM episodes over a median follow-up period of mean 5.4 years. Table 1 shows the general characteristics of the participants among incident T2DM according to gender. In both the male and female groups, the occurrence of T2DM was associated with older age, higher smoking status, higher baseline levels of ALT, AST, GGT, TC, TG, HbA1c, FPG, SBP, DBP, weight, BMI, WC, WHtR, FM, LM, PF, lower baseline level of HDL-c and height. At baseline, there was a statistically significant association with exercise and alcohol consumption habits in the men group but not in the women group. Moreover, the average FM and PF in men was significantly higher than that in women (24.6 ± 5.9 vs. 9.0 ± 4.6, 35.8 ± 3.2 vs. 20.3 ± 3.4, *p* value < 0.001), LM is slightly higher in women than in men(42.1 ± 4.1 vs. 41.3 ± 4.3, *p* value < 0.001) Table S2 ( supplemental table S2).

**Table 1:** Clinical characteristics of the study population

		Men		Women		
Variables	Non - T2DM (*n* = 8133)	T2DM(*n* = 286)	*p* value	Non - T2DM (*n* = 6947)	T2DM(*n* = 87)	*p* value
Age, (years)	44.0 ± 9.0	47.0 ± 8.5	< 0.001	43.2 ± 8.7	47.6 ± 8.5	< 0.001
Habit of exercise, n(%)			0.019			0.611
No	6575 (80.8)	247 (86.4)		5850 (84.2)	75 (86.2)	
Yes	1558 ( 19.2)	39 ( 13.6)		1097 ( 15.8)	12 ( 13.8)	
Alcohol consumption, n(%)			0.018			0.394
None	5167 (63.5)	184 (64.3)		6369 (91.7)	82 (94.3)	
Light	1327 ( 16.3)	38 ( 13.3)		387 (5.6)	2 (2.3)	
Moderate	1129 ( 13.9)	34 ( 11.9)		191 (2.7)	3 (3.4)	
Heavy	510 (6.3)	30 ( 10.5)		0	0	
Smoking status, n(%)			< 0.001			0.02
Never	2813 (34.6)	75 (26.2)		6069 (87.4)	70 (80.5)	
Past	2436 (30)	72 (25.2)		436 (6.3)	5 (5.7)	
Current	2884 (35.5)	139 (48.6)		442 (6.4)	12 ( 13.8)	
ALT, (IU/L)	20.0 (15.0, 27.0)	28.0 (20.0, 42.8)	< 0.001	14.0 (11.0, 17.0)	19.0 (14.0, 23.0)	< 0.001
AST, (IU/L)	18.0 (15.0, 23.0)	20.5 (17.0, 26.8)	< 0.001	16.0 (13.0, 19.0)	18.0 (15.0, 22.0)	< 0.001
GGT, (IU/L)	19.0 (15.0, 28.0)	26.0 (19.0, 39.8)	< 0.001	12.0 (10.0, 15.0)	15.0 (12.0, 22.5)	< 0.001
HDL-c, (mg/dL)	50.7 ± 13.4	43.5 ± 11.5	< 0.001	63.9 ± 14.8	53.9 ± 13.3	< 0.001
TC, (mg/dL)	198.0 (176.0, 220.0)	210.0 (185.0, 229.0)	< 0.001	194.0 (172.0, 218.0)	212.0 (188.5, 241.5)	< 0.001
TG, (mg/dL)	81.0 (56.0, 121.0)	125.5 (80.5, 189.0)	< 0.001	50.0 (36.0, 71.0)	85.0 (64.5, 115.5)	< 0.001
HbA1c, (%)	5. 1 ± 0.3	5.5 ± 0.4	< 0.001	5.2 ± 0.3	5.6 ± 0.4	< 0.001
FPG, (mg/dL)	95.4 ± 6.6	101.9 ± 5.9	< 0.001	89.7 ± 7.0	98.6 ± 7.4	< 0.001
SBP, (mmHg)	118.6 ± 14. 1	123.5 ± 15.5	< 0.001	109.3 ± 14.3	117.0 ± 15.0	< 0.001
DBP, (mmHg)	74.7 ± 9.9	78.5 ± 10.2	< 0.001	67.6 ± 9.8	72.9 ± 9.3	< 0.001
BodyWeight, (kg)	67. 1 ± 9.8	73.0 ± 12.3	< 0.001	52.6 ± 7.8	59.5 ± 11. 1	< 0.001
Height, (cm)	170.8 ± 6.0	170.0 ± 6.0	0.016	158.3 ± 5.4	155.9 ± 6.5	< 0.001
BMI, (kg/m2)	23.0 ± 2.9	25.2 ± 3.6	< 0.001	21.0 ± 2.9	24.5 ± 4.4	< 0.001
WC, (cm)	80.3 ± 7.8	86.5 ± 9.2	< 0.001	71.6 ± 8.0	80.4 ± 11.8	< 0.001
WHtR	47.0 ± 4.6	50.9 ± 5.2	< 0.001	45.2 ± 5. 1	51.6 ± 7.7	< 0.001
FM	24.4 ± 5.8	28.6 ± 7.2	< 0.001	9.0 ± 4.5	14. 1 ± 6.8	< 0.001
LM	41.3 ± 4.2	43.0 ± 5.2	< 0.001	42. 1 ± 4. 1	43.9 ± 5.3	< 0.001
PF	35.7 ± 3. 1	38.4 ± 3.5	< 0.001	20.2 ± 3.3	24. 1 ± 4.9	< 0.001

Data were mean ± SD or median (IQR) for continuous variables or numbers (proportions) for categorical variables

*ALT* alanine aminotransferase, *AST* aspartate aminotransferase, *GGT* gamma glutamyl transferase, *HDL-c* high‐density lipoprotein cholesterol, *TC* total cholesterol, *TG* triglyceride, *HbA1c* hemoglobin A1c, *FPG* fasting plasma glucose, *SBP* systolic blood pressure, *DBP* diastolic blood pressure, *BMI* body mass index, *WC* waist circumference, *WHtR* waist‐‐height ratio, *T2DM* type 2 diabetes mellitus, *FM* predicted fat mass, *LM* predicted lean mass, *PF* predicted per cent fat, Q1, Q2, Q3 are tertiles of the predicted FM, LM, PF

### Univariate and multivariate Cox regression analyses of Incident T2DM

Table S3 (supplementary table S3) displays the results of a univariable Cox regression analysis. Variables that were statistically significant in univariable analysis (*p* value < 0.1), the matched odds ratio changed at least 10% if it was added to this model or clinically relevant were included in the multivariable analysis. We gradually adjusted for the following confounding factors: age, habit of exercise, smoking status, alcohol consumption, ALT, AST, GGT, HDL, TC, TG, HbA1c, FPG, SBP, DBP levels. As shown in Table 2, when the predicted FM, LM and PF were assessed separately as continuous variables, an increase in the risk of developing T2DM was observed in the male group after adjustment for multivariate (HR = 1.05, 95%CI: 1.03 ~ 1.07, HR = 1.04, 95%CI: 1.01 ~ 1.07, HR = 1.11, 95%CI: 1.07 ~ 1.16, respectively, each *p*-value < 0.05 ). All the predicted FM, LM, and PF were then divided into tertiles. When predicted FM, LM and PF are evaluated separately as categorical variables, the adjusted HRs were 1, 0.82 (95%CI: 0.56 ~ 1.19), 1.25 (95%CI: 0.88 ~ 1.79) across predicted FM tertiles (*p*-value: 0.077); 1, 0.76 (95%CI: 0.54 ~ 1.07), 1.13 (95%CI: 0.83 ~ 1.56) across predicted LM tertiles (*p*-value: 0.287); 1, 1.03 (95%CI: 0.67 ~ 1.58), 1.52 (95%CI: 0.99 ~ 2.33) across predicted PF tertiles (*p*-value: 0.01). In the female group, the risk of T2DM increased with increasing predicted FM and PF. Being at the top predicted FM and PF tertile groups remained significantly associated with T2DM after multiple-factor correction (HR = 2.52, 95%CI: 1.15 ~ 5.55, HR = 2.21, 95%CI: 1.03 ~ 4.73, respectively, both *p* value < 0.05). However, no correlation was found for predicted LM and T2DM in the female group (HR = 1.03, 95%CI: 0.98 ~ 1.07, *p* value: 0.274).

**Table 2:** Results of multivariate Cox regression analysis of correlation between FM, LM, PF and T2DM

Variable	Unadjusted		Model 1		Model 2		Model 3	
	HR(95%CI)	* p* value	HR(95%CI)	* p* value	HR(95%CI)	*p* value	HR(95%CI)	*p* value
Men								
FM	1. 1 ( 1.08 ~ 1. 11)	< 0.001	1. 11 ( 1.09 ~ 1. 13)	< 0.001	1. 11 ( 1.09 ~ 1. 12)	< 0.001	1.05 ( 1.03 ~ 1.07)	< 0.001
1st tertile(< 21.783)	1(Ref)		1(Ref)		1(Ref)		1(Ref)	
2st tertile(21.783 ~ 26.335)	1.28 (0.89 ~ 1.85)	0. 183	1.23 (0.85 ~ 1.77)	0.271	1.28 (0.88 ~ 1.84)	0. 192	0.82 (0.56 ~ 1. 19)	0.29
3st tertile(> 26.335)	3.43 (2.51 ~ 4.7)	< 0.001	3.47 (2.53 ~ 4.74)	< 0.001	3.57 (2.61 ~ 4.89)	< 0.001	1.25 (0.88 ~ 1.79)	0.211
*p* for trend		< 0.001		< 0.001		< 0.001		0.077
LM	1. 1 ( 1.07 ~ 1. 12)	< 0.001	1. 13 ( 1. 11 ~ 1. 16)	< 0.001	1. 13 ( 1. 1 ~ 1. 16)	< 0.001	1.04 ( 1.01 ~ 1.07)	0.006
1st tertile(< 39.37)	1(Ref)		1(Ref)		1(Ref)		1(Ref)	
2st tertile(39.37 ~ 42.81)	0.84 (0.61 ~ 1. 16)	0.296	1.02 (0.73 ~ 1.42)	0.909	1.05 (0.75 ~ 1.47)	0.766	0.76 (0.54 ~ 1.07)	0. 112
3st tertile(> 42.81)	1.87 ( 1.42 ~ 2.46)	< 0.001	2.59 ( 1.93 ~ 3.47)	< 0.001	2.6 ( 1.95 ~ 3.49)	< 0.001	1. 13 (0.83 ~ 1.56)	0.434
*p* for trend		< 0.001		< 0.001		< 0.001		0.287
PF	1.23 ( 1.2 ~ 1.26)	< 0.001	1.24 ( 1.2 ~ 1.27)	< 0.001	1.23 ( 1.2 ~ 1.27)	< 0.001	1. 11 ( 1.07 ~ 1. 16)	< 0.001
1st tertile(< 34.343)	1(Ref)		1(Ref)		1(Ref)		1(Ref)	
2st tertile(34.343 ~ 36.976)	2.24 ( 1.49 ~ 3.37)	< 0.001	2.04 ( 1.35 ~ 3.08)	0.001	2.08 ( 1.38 ~ 3. 13)	< 0.001	1.03 (0.67 ~ 1.58)	0.895
3st tertile(> 36.976)	5.63 (3.88 ~ 8. 17)	< 0.001	4.88 (3.34 ~ 7. 12)	< 0.001	5.01 (3.43 ~ 7.3)	< 0.001	1.52 (0.99 ~ 2.33)	0.055
*p* for trend		< 0.001		< 0.001		< 0.001		0.01
Women								
FM	1.2 ( 1. 16 ~ 1.23)	< 0.001	1.2 ( 1. 16 ~ 1.23)	< 0.001	1. 19 ( 1. 16 ~ 1.23)	< 0.001	1. 11 ( 1.07 ~ 1. 16)	< 0.001
1st tertile(< 6.717)	1(Ref)		1(Ref)		1(Ref)		1(Ref)	
2st tertile (6.717 ~ 10.356)	2.52 ( 1. 13 ~ 5.63)	0.024	2.39 ( 1.07 ~ 5.34)	0.033	2.37 ( 1.06 ~ 5.28)	0.036	1.76 (0.78 ~ 3.98)	0. 175
3st tertile (> 10.356)	8.72 (4.32 ~ 17.61)	< 0.001	7.45 (3.68 ~ 15. 1)	< 0.001	7.53 (3.71 ~ 15.28)	< 0.001	2.52 ( 1. 15 ~ 5.55)	0.022
*p* for trend		< 0.001		< 0.001		< 0.001		0.018
LM	1.08 ( 1.03 ~ 1. 13)	0.001	1. 11 ( 1.06 ~ 1. 16)	< 0.001	1. 1 ( 1.05 ~ 1. 16)	< 0.001	1.03 (0.98 ~ 1.07)	0.274
1st tertile(< 40.075)	1(Ref)		1(Ref)		1(Ref)		1(Ref)	
2st tertile(40.075 ~ 43.432)	0.94 (0.52 ~ 1.69)	0.83	1.06 (0.58 ~ 1.92)	0.853	1.01 (0.56 ~ 1.84)	0.965	1. 1 (0.6 ~ 2.02)	0.77
3st tertile(> 43.432)	1.64 (0.97 ~ 2.78)	0.062	2.02 ( 1. 19 ~ 3.44)	0.01	2.01 ( 1. 18 ~ 3.42)	0.011	1.4 (0.79 ~ 2.49)	0.254
*p* for trend		0.038		0.006		0.006		0.236
PF	1.31 ( 1.26 ~ 1.37)	< 0.001	1.3 ( 1.24 ~ 1.36)	< 0.001	1.3 ( 1.24 ~ 1.36)	< 0.001	1. 18 ( 1. 11 ~ 1.26)	< 0.001
1st tertile(< 18.47)	1(Ref)		1(Ref)		1(Ref)		1(Ref)	
2st tertile(18.47 ~ 21.412)	2.52 ( 1. 17 ~ 5.44)	0.019	2.39 ( 1. 11 ~ 5. 16)	0.026	2.4 ( 1. 11 ~ 5. 18)	0.026	1.58 (0.72 ~ 3.48)	0.255
3st tertile(> 21.412)	9 (4.57 ~ 17.72)	< 0.001	7.34 (3.7 ~ 14.56)	< 0.001	7.4 (3.72 ~ 14.7)	< 0.001	2.21 ( 1.03 ~ 4.73)	0.041
*p* for trend		< 0.001		< 0.001		< 0.001		0.033

Model 1 adjust for age

Model 2 adjust for Model 1+habit of exercise, smoking status and alcohol consumption

Model 3 adjust for Model 1+Model 2+ALT, AST, GGT, HDL, TC, TG, HbA1c, FBP, SBP and DBP levels

*Ref *reference

### Nonlinear and linear relationship exploration

**Fig. 1:**
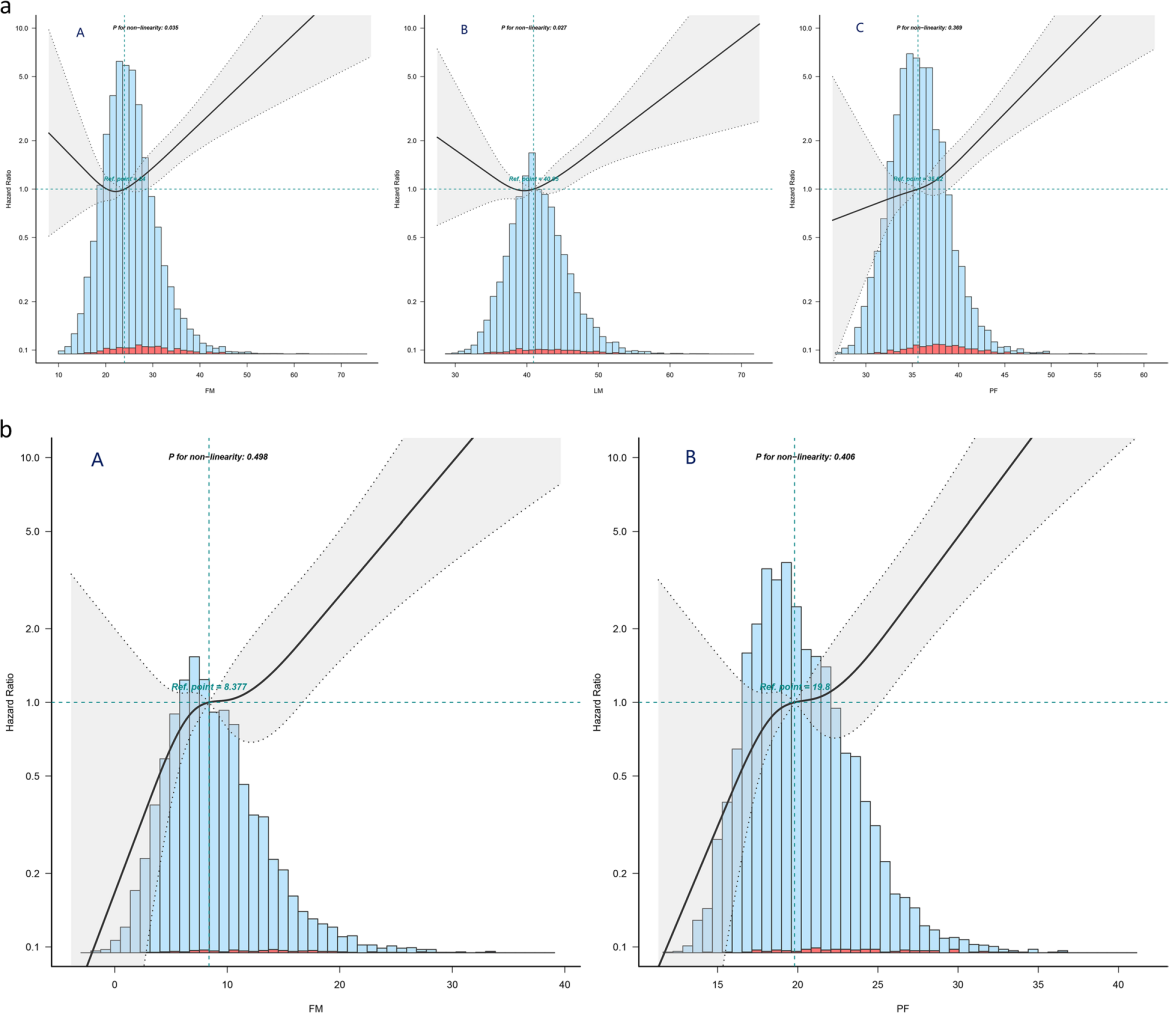
** a** The association between predicted fat mass (FM, A), predicted lean mass (LM, B), predicted percent fat (PF, C) and T2DM events in male participants. The solid line indicates the estimated risk of T2DM incidence, and the shadow represents point wise 95% CI after adjusting for age, habit of exercise, smoking status, alcohol consumption, alanine aminotransferase (ALT), aspartate aminotransferase (AST), g-glutamyl transferase (GGT), high-density lipoprotein cholesterol (HDL), total cholesterol (TC), triglyceride (TG), hemoglobin A1c (HbA1c), fasting glucose (FPG), systolic blood pressure (SBP) and diastolic blood pressure (DBP) levels. **b** The association between predicted fat mass (FM, A), predicted percent fat (PF, B) and T2DM events in female participants. The solid line indicates the estimated risk of T2DM incidence, and the shadow represents point wise 95% CI after adjusting for age, habit of exercise, smoking status, alcohol consumption, alanine aminotransferase (ALT), aspartate aminotransferase (AST), g-glutamyl transferase (GGT), high-density lipoprotein cholesterol (HDL), total cholesterol (TC), triglyceride (TG), hemoglobin A1c (HbA1c), fasting glucose (FPG), systolic blood pressure (SBP) and diastolic blood pressure (DBP) levels

In men group, the relationship between predicted FM, LM and T2DM risk was non-linear (*p* value, non-linearity < 0.05), and the model using restricted cubic splines with four knots showed a U-shaped association (Fig. 1a). The lowest and highest tertiles of predicted FM, LM were associated with an increased T2DM onset risk in men group. The probability of developing T2DM was lowest in the moderate tertiles of predicted FM and LM. We discovered a linear relationship between men’s predicted PF and risk of developing of T2DM. Figure 1b shows the linear association between the predicted FM, PF and risk of developing T2DM in women. As predicted FM and PF increased, so did the probability of T2DM in women.

### Subgroup analyses by adjusted potential effect confounders

**Fig. 2:**
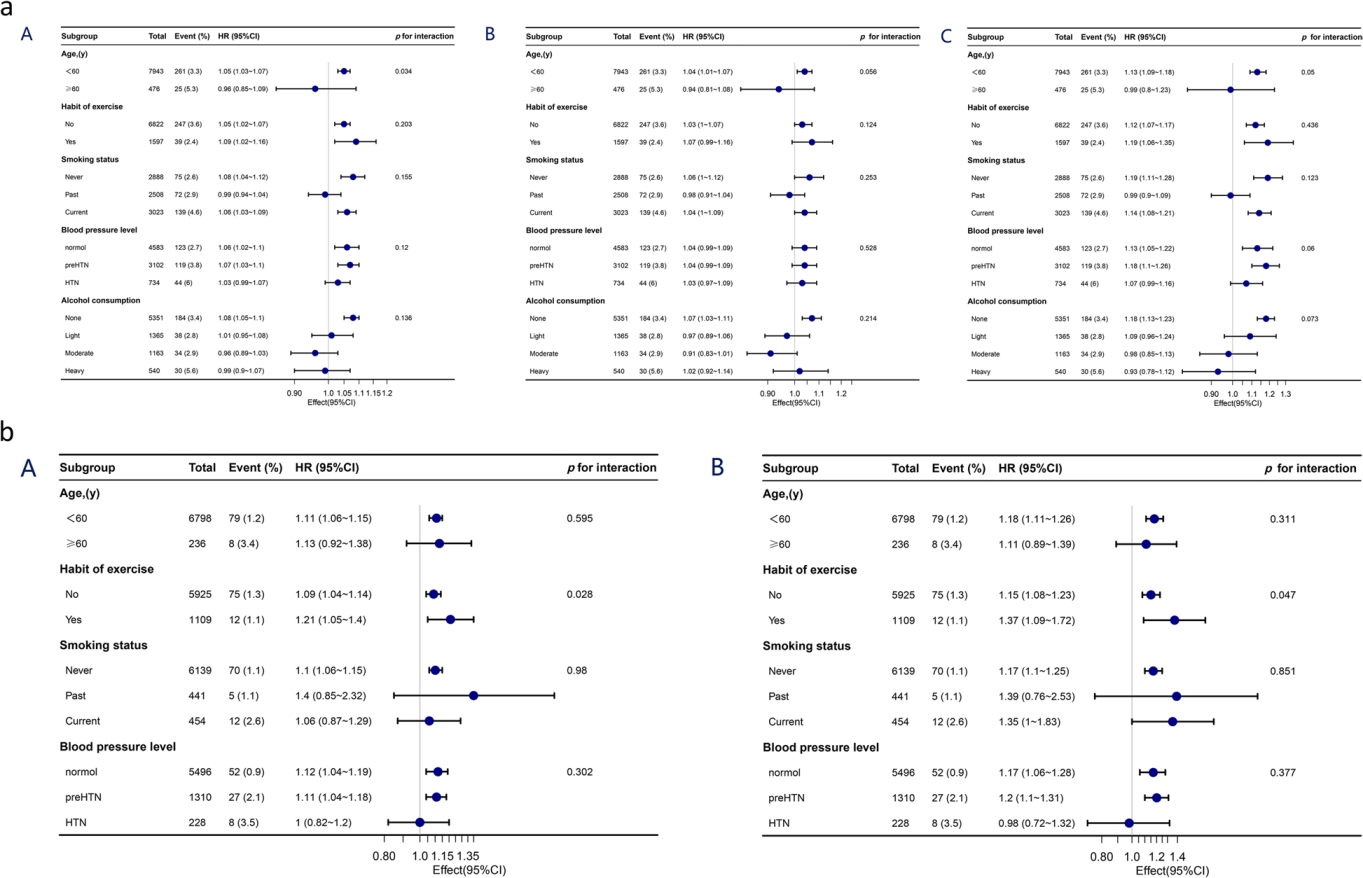
**a** Subgroup analyses of the predicted FM (A), predicted LM (B), predicted PF (C) and T2DM risk in male participants. **b** Subgroup analyses of the predicted FM (A), predicted PF (B) and T2DM risk in female participants

The robustness of our findings was evaluated using the subgroup sensitivity analysis. Age, habit of exercise, smoking status, alcohol consumption, ALT, AST, GGT, HDL, TC, TG, HbA1c, FPG, SBP, DBP levels were all adjusted in this analysis. The estimated risk of predicted FM, LM, PF, and T2DM was more closely associated with younger age in males and exercise habit in women. In the subgroup analysis, no other noteworthy interactions were found (Fig. 2a, b).

### Relationship of predicted FM, LM and PF with the risk of T2DM

During a median period of 5.4 years [interquartile range (IQR): 2.7–9.4 years], a total of 373 T2DM occurred [median (IQR) time to T2DM: 5.9 (3.0-8.9) years), including 286 men and 87 women. Each of these three factors, including predicted FM, LM and PF, was divided into three groups, ranging from low (tertile 1) to high (tertile 3). Kaplan-Meier curves showed that both men and women participants in tertile group 3 of predicted FM, LM and PF had the highest cumulative incidence of T2DM probability, while those in tertile group 1 of predicted FM, LM, and PF had the lowest cumulative incidence of T2DM. Figure S2a, b (supplemental figure S2a, b).

### Predicting value of predicted FM, LM, PF in Incident T2DM

BMI, WC, and WHtR were all significantly correlated with T2DM risk in our study Table S4 (supplemental table S4). Table 3 shows the results of ROC analysis, in which the area under the curve (AUC) for predicted FM, LM, PF in men was 0.673 (95%CI: 0.639 ~ 0.707), 0.598 (95%CI: 0.561 ~ 0.635), 0.715 (95%CI: 0.684 ~ 0.745), respectively. In men group, WHtR and predicted PF had the higher Harrell’s c-index (0.7151, 0.7150, respectively), which exhibited the larger AUC compared with BMI and WC (*p*-value < 0.05). The AUC for predicted FM and PF in the group of women was 0.739 (95%CI: 0.683 ~ 0.796) and 0.742 (95%CI: 0.687 ~ 0.798), respectively. WHtR was found to have the strongest correlation, followed by BMI, predicted PF, WC, and predicted FM.

**Table 3:** Predictive performance of predict FM, LM, PF, BMI, WC and WHtR for incident T2DM

Variables	Men		women	
	Harrell’s c-index	95%CI	Harrell’s c-index	95%CI
FM	0.673	0.639 to 0.707	0.739	0.683 to 0.796
LM	0.598	0.561 to 0.635	-	-
PF	0.7150	0.684 to 0.745	0.742	0.687 to 0.798
BMI	0.684	0.651 to 0.717	0.757	0.704 to 0.811
WC	0.700	0.666 to 0.733	0.733	0.676 to 0.789
WHtR	0.7151	0.684 to 0.746	0.758	0.703 to 0.813

*FM *predicted fat mass, *LM *predicted lean mass, *PF *predicted per cent fat;

*BMI* body mass index, *WC* waist circumference, *WHtR* waist-to-height ratio;

*CI* confidence interval

## Discussion

In the current study, we analyzed the strength of associations between three novel body composition measurements, including predicted FM, LM, PF, and risk of T2DM among Japanese adults, and assessed the strength of relationships with existing obesity markers. Predicted FM, LM, and PF were all significantly associated with a greater risk of developing new-onset T2DM in the male group. A U-shaped relationship between predicted FM, LM, and T2DM onset was discovered in adult male Japanese subjects. Other commonly used markers of male obesity performed worse in discrimination than WHtR and predicted PF. The risk of T2DM was individually and linearly correlated with predicted FM and PF in the female group, while the greatest predictors were WHtR and BMI.

### Clinical implications

T2DM is a growing epidemic and associated with severe cardiovascular complications. The increasing prevalence of adults with T2DM has become a major social burden which due to population aging, the rising prevalence of risk factors such obesity, physical inactivity, and bad eating habits [14]. To halt the T2DM epidemic, early detection of diabetic and prediabetic patients is very important. Therefore, there is an urgent need for a simple, reliable, practical and inexpensive metric to assess the risk of developing T2DM. Despite the fact that BMI is frequently employed as a measure of obesity, several research have discovered that it does not account for the distribution of adipose tissue, lean mass, or fat mass [15]. In order to more accurately depict body composition, recent studies have created equations that predict FM, LM, and PF [12]. The prediction equation is simple to calculate, requiring only information such as gender, race, height, weight, and waist circumference, which are easily measured and available. Recently, in two sizable US prospective cohorts, Lee and colleagues [6] examined the relationship between predicted FM and T2DM risk. The magnitude of the association between BMI and other obesity indicators was compared. They discovered that among men, predicted FM had the highest correlation with T2DM. For women, the WHtR was the strongest indicator, followed by WC, predicted PF, predicted FM, BMI. Of note, predicted FM demonstrated a stronger association with T2DM risk than BMI. A 15-year prospective cohort from China [16], recruited 711 people from the general population, founding that the predicted PF, LM, PF could independently predict T2DM risk for Chinese males. Compared to other commonly used obesity indicators, predicted FM performed better in discrimination. In contrast, the association was not found in Chinese females. Consistent with previous studies, we also found the association between predicted FM, LM, PF and T2DM risk in Japanese adults. While, some results are partly different from our findings. In the present study, we found a U-shaped relationship between predicted FM, LM and T2DM onset in Japanese adult men. Those individuals in the middle tertile group had the lowest risk of new-onset T2DM. In other words, predicted FM, LM levels that are too high or too low are associated with an increased risk of T2DM. However, no similar relationship was found in previous research in other populations. The possible reasons for the different results in the studies may due to differential sample sizes, ethnicity, cultural background and covariates being adjusted.

Although the molecular causes of obesity and T2DM are still not fully understood, chronic inflammation, particularly in adipose tissue, may be a factor [17, 18]. Sentinel events in metabolic dysregulation associated with obesity include adipocyte lipid storage malfunction and adipose tissue insulin resistance. Increased visceral fat in obese teens is linked to impaired lipogenic or adipogenic capacity [19], and lower omental adipocyte size in obese adults is linked to metabolic health [20]. One may argue that the increased mass of adipose tissue is a state of increased inflammatory mass since an estimated excess of 20–30 million macrophages accumulate with each kilogram of extra fat in individuals [21]. Insulin sensitivity may be affected by adipokines (inflammatory molecules secreted by AT) in various tissues, particularly skeletal muscle and liver, by causing inflammation in the adipose tissue and contributing to whole body insulin resistance. The body’s most crucial organ for maintaining glucose homeostasis is the skeletal muscle [22], which, under normal circumstances, is in charge of 80–90% of the body’s insulin-stimulated glucose absorption and excretion [17, 23]. Insulin resistance in skeletal muscle is the main defect in T2DM and is therefore central to systemic insulin resistance and T2DM [23]. As the main tissue target for insulin action and a major contributor to insulin resistance, lean body mass (primarily skeletal muscle) may play a beneficial role for reducing risk of T2DM [24, 25]. In this study, we more accurately distinguished between fat mass, lean mass, and percent fat using proven anthropometric prediction algorithms. In the study, predicted PF was found to be more strongly related to men’s T2DM risks than BMI. This result also indicates that the negative effects of fat mass on T2DM risk [26] are not fully reflected by BMI, and this limitation may be somewhat overcome by predicted percent fat, which were in line with the results of earlier studies [6].

Numerous research have looked at the connection between obesity and a number of indicators, each of which represents a distinct element of body composition, despite the fact that there is no clear agreement on which indicator better predicts T2DM. For instance, in the Indian population, WC and WHtR show a greater correlation with T2DM than BMI [27]. Compared to BMI and WHtR, WC is a straightforward and reliable indicator of T2DM in the Chinese population [28]. Another study in the Chinese population found that WHtR performed better for the connection with T2DM than BMI and WC [29]. However, the results of a meta-analysis of prospective studies did not find any significant difference in the development of T2DM by WC, WHR and BMI [30]. Recent studies suggested that ectopic fat obesity may present the largest risk of incident T2DM rather than overall adiposity [4]. In our study, the incidence rates of T2DM vary significantly between different genders, which is more common in men than women. Meanwhile, certain obesity indicators vary between men and women. We found that the WHtR has the strongest correlation with T2DM risk in both genders. In the men group, predicted PF and WC measures were more strongly associated with risk of T2DM than BMI. In other words, our study reveals that among Japanese men adults, obesity measures that better reflect absolute or relative quantities of adiposity, such as WHtR, predicted PF and WC, may have a greater connection with T2DM risk than BMI. Whereas in the women, unlike men, BMI was more accurate than predicted PF and WC in predicting T2DM.

### Limitations

As far as we know, in the present study, we show for the first time to investigate the predictive power of three new body composition parameters on the risk of T2DM in a retrospective cohort in Japan. The study’s strengths include the use of a standardized lifestyle questionnaire, and a relatively large longitudinal study based on a population-based sample. Some limitations also exist in our study. First, due to the limited amount of data available in this secondary analysis, it was unable to fully adjust for unmeasured or unpublished confounding variables in original study. We known that lifestyle, and particularly food habit, is one of the important factor in obesity as risk factor to predict T2DM. We will consider this limitation in our further research. Second, the T2DM incidence rate might have been underestimated since oral glucose tolerance tests were not used in the subsequent investigation. Third, all formulas used were based on predictions, which may not fully capture the phenomena of adiposity and lean mass among the population. Last, it is uncertain whether our study can be generalized to non-Japanese populations.

## Conclusion

In conclusion, T2DM risk in Japanese individuals was substantially correlated with predicted FM, LM, and PF. However, more comprehensive researches are needed to identify the appropriate obesity parameters to predict T2DM in Japanese adults, especially among different genders. Testing the reliability of predicted PF, FM, and LM in assessing T2DM in additional racial/ethnic populations and their applicability in clinical settings would be beneficial.

### Supplementary Information

**Supplementary Material 1.**

## Data Availability

The raw data can be downloaded from the ‘DATADRYAD’ database (www.Datadryad.org). Dryad Digital Repository. https://datadryad.org/stash/dataset/doi:10.5061%2Fdryad.8q0p192.
