# Supplementary material for: Association between predicted fat mass, predicted lean mass, predicted percent fat and type 2 diabetes mellitus in Japanese adults: a retrospective study
Source: BMC Endocr Disord. 2024 Apr 17;24:48. doi: 10.1186/s12902-024-01579-4 (PMC11022471; doi:10.1186/s12902-024-01579-4)
Supplement: Supplementary file 1 — Supplementary Material 1. [file 12902_2024_1579_MOESM1_ESM.docx]

Table S1

**Equation profiles**

**Equations for predicted fat mass (FM, kg)**

For men = −18.592-0.009 × age (year) − 0.080 × height (cm) + 0.226 × weight (kg)+ 0.387 × WC (cm) +0.080 [if Mexican] - 0.188 [if Hispanic] - 0.483 [if Black] + 1.050 [if other race]

For women = 11.817 + 0.041 × age (year) − 0.199 × height (cm) + 0.610 × weight (kg)+ 0.044 × WC (cm) +0.338 [if Mexican] + 0.073 [if Hispanic] - 1.187 [if Black] + 0.325 [if other race]

**Equations for predicted lean mass (LM, kg)**

For men = 19.363 + 0.001 × age (year) + 0.064 × height (cm) + 0.756 × weight (kg)− 0.366 × WC (cm) - 0.066 [if Mexican] + 0.231 [if Hispanic] + 0.432 [if Black] - 1.007 [if other race]

For women = −10.683 − 0.039 × age (year) + 0.186 × height (cm) + 0.383 × weight (kg)− 0.043 × WC (cm) - 0.359 [if Mexican] - 0.059 [if Hispanic] + 1.085 [if Black] - 0.34 [if other race]

**Equations for predicted percent fat (PF, %)**

For men = 0.02 + 0.00 × age (year) − 0.07 × height (cm) − 0.08 × weight (kg)+ 0.48 × WC (cm) + 0.32 [if Mexican] + 0.02 [if Hispanic] - 0.65 [if Black] +1.12 [if other race]

For women = 50.46 + 0.07 × age (year) − 0.26 × height (cm) + 0.27 × weight (kg)+ 0.10 × WC (cm) + 0.89 [if Mexican] + 0.49 [if Hispanic] - 1.57 [if Black] + 0.43 [if other race]

Table S2 Clinical characteristics of the study population.

| Variables | Total (n = 15453) | Men (n = 8419) | Women(n = 7034) | *p* value |
| --- | --- | --- | --- | --- |
| Age,(years) | 43.7 ± 8.9 | 44.1 ± 9.0 | 43.3 ± 8.8 | < 0.001 |
| Habit of exercise,n(%) |  |  |  | < 0.001 |
| No | 12747 (82.5) | 6822 (81) | 5925 (84.2) |  |
| Yes | 2706 (17.5) | 1597 (19) | 1109 (15.8) |  |
| Alcohol consumption,n(%) |  |  |  | < 0.001 |
| None | 11802 (76.4) | 5351 (63.6) | 6451 (91.7) |  |
| Light | 1754 (11.4) | 1365 (16.2) | 389 (5.5) |  |
| Moderate | 1357 ( 8.8) | 1163 (13.8) | 194 (2.8) |  |
| Heavy | 540 ( 3.5) | 540 (6.4) | 0 (0) |  |
| Smoking status,n(%) |  |  |  | < 0.001 |
| Never | 9027 (58.4) | 2888 (34.3) | 6139 (87.3) |  |
| Past | 2949 (19.1) | 2508 (29.8) | 441 (6.3) |  |
| Current | 3477 (22.5) | 3023 (35.9) | 454 (6.5) |  |
| ALT,(IU/L) | 17.0 (13.0, 23.0) | 20.0 (16.0, 28.0) | 14.0 (11.0, 17.0) | < 0.001 |
| AST,(IU/L) | 17.0 (14.0, 21.0) | 18.0 (15.0, 23.0) | 16.0 (13.0, 19.0) | < 0.001 |
| GGT,(IU/L) | 15.0 (11.0, 22.0) | 20.0 (15.0, 29.0) | 12.0 (10.0, 15.0) | < 0.001 |
| HDL-c,(mg/dL) | 56.5 ± 15.6 | 50.5 ± 13.4 | 63.8 ± 14.9 | < 0.001 |
| TC,(mg/dL) | 196.0 (174.0, 219.0) | 198.0 (176.0, 220.0) | 194.0 (172.0, 218.0) | < 0.001 |
| TG,(mg/dL) | 65.0 (44.0, 99.0) | 82.0 (56.0, 123.0) | 50.0 (36.0, 72.0) | < 0.001 |
| HbA1c,(%) | 5.2 ± 0.3 | 5.2 ± 0.3 | 5.2 ± 0.3 | < 0.001 |
| FPG,(mg/dL) | 93.0 ± 7.4 | 95.6 ± 6.7 | 89.8 ± 7.1 | < 0.001 |
| SBP,(mmHg) | 114.5 ± 15.0 | 118.8 ± 14.1 | 109.4 ± 14.3 | < 0.001 |
| DBP,(mmHg) | 71.6 ± 10.5 | 74.9 ± 10.0 | 67.6 ± 9.8 | < 0.001 |
| BodyWeight,(kg) | 60.6 ± 11.6 | 67.3 ± 9.9 | 52.7 ± 7.9 | < 0.001 |
| Height,(cm) | 165.1 ± 8.5 | 170.8 ± 6.0 | 158.3 ± 5.4 | < 0.001 |
| BMI,(kg/m2) | 22.1 ± 3.1 | 23.0 ± 3.0 | 21.0 ± 2.9 | < 0.001 |
| WC,(cm) | 76.5 ± 9.1 | 80.5 ± 7.9 | 71.7 ± 8.1 | < 0.001 |
| WHtR | 0.5 ± 0.0 | 0.5 ± 0.0 | 0.5 ± 0.1 | < 0.001 |
| FM | 17.5 ± 9.4 | 24.6 ± 5.9 | 9.0 ± 4.6 | < 0.001 |
| LM | 41.7 ± 4.2 | 41.3 ± 4.3 | 42.1 ± 4.1 | < 0.001 |
| PF | 28.7 ± 8.4 | 35.8 ± 3.2 | 20.3 ± 3.4 | < 0.001 |
| Follow up duration,(years) | 5.4 (2.7, 9.4) | 5.8 (2.8, 9.9) | 5.1 (2.6, 9.0) | < 0.001 |
| Incident T2DM,n(%) |  |  |  | < 0.001 |
| No | 15080 (97.6) | 8133 (96.6) | 6947 (98.8) |  |
| Yes | 373 ( 2.4) | 286 (3.4) | 87 (1.2) |  |

Data were mean ± SD or median (IQR) for continuous variables or numbers (proportions) for categorical variables.

ALT, alanine aminotransferase; AST, aspartate aminotransferase; GGT, gamma glutamyl transferase; HDL-c, high‐density lipoprotein cholesterol; TC, total cholesterol; TG, triglyceride; HbA1c, hemoglobin A1c; FPG, fasting plasma glucose; SBP, systolic blood pressure; DBP, diastolic blood pressure; BMI, body mass index; WC, waist circumference; WHtR, waist‐‐height ratio; T2DM, type 2 diabetes mellitus; FM, fat mass; LM, lean mass; PF, per cent fat;

Table S3 Results of univariate Cox regression analysis of T2DM.

|  | men | |  | women | |
| --- | --- | --- | --- | --- | --- |
| Variables | HR(95%CI) | *p* value |  | HR(95%CI) | *p* value |
| Age,(years) | 1.05 (1.03,1.06) | < 0.001 |  | 1.08 (1.05,1.11) | < 0.001 |
| Habit of exercise Yes vs No | 0.71 (0.51,1) | 0.041 |  | 0.83 (0.45,1.52) | 0.532 |
| Alcoholconsumption |  |  |  |  |  |
| None | ref | 0.019 |  | ref | 0.254 |
| Light | 0.73 (0.51,1.03) |  |  | 0.39 (0.1,1.59) |  |
| Moderate | 0.81 (0.56,1.17) |  |  | 1.42 (0.45,4.51) |  |
| Heavy | 1.52 (1.03,2.24) |  |  | 0 |  |
| Smokingstatus |  |  |  |  |  |
| Never | ref | < 0.001 |  | ref | 0.009 |
| Past | 1.11 (0.8,1.54) |  |  | 1.35 (0.55,3.36) |  |
| Current | 1.69 (1.27,2.23) |  |  | 2.93 (1.59,5.42) |  |
| ALT,(IU/L) | 1.02 (1.02,1.03) | < 0.001 |  | 1.0049 (1.0022,1.0077) | 0.032 |
| AST,(IU/L) | 1.03 (1.03,1.04) | < 0.001 |  | 1.0068 (1.0023,1.0114) | 0.064 |
| GGT,(IU/L) | 1.0086 (1.0059,1.0112) | < 0.001 |  | 1.03 (1.02,1.03) | < 0.001 |
| HDL-c,(mg/dL) | 0.95 (0.94,0.97) | < 0.001 |  | 0.95 (0.93,0.97) | < 0.001 |
| TC,(mg/dL) | 1.008 (1.0046,1.0113) | < 0.001 |  | 1.02 (1.01,1.02) | < 0.001 |
| TG,(mg/dL) | 1.0054 (1.0045,1.0064) | < 0.001 |  | 1.01 (1.01,1.01) | < 0.001 |
| HbA1,(%) | 53.29 (37.1,76.55) | < 0.001 |  | 79.48 (40.49,156.01) | < 0.001 |
| FPG,(mg/dL) | 1.2 (1.17,1.22) | < 0.001 |  | 1.2 (1.16,1.23) | < 0.001 |
| SBP,(mmHg) | 1.03 (1.02,1.03) | < 0.001 |  | 1.03 (1.02,1.04) | < 0.001 |
| DBP,(mmHg) | 1.04 (1.03,1.05) | < 0.001 |  | 1.05 (1.03,1.07) | < 0.001 |
| BodyWeight,(kg) | 1.05 (1.04,1.06) | < 0.001 |  | 1.09 (1.07,1.11) | < 0.001 |
| Height,(cm) | 0.98 (0.96,1) | 0.021 |  | 0.92 (0.89,0.96) | < 0.001 |
| BMI,(kg/m2) | 1.21 (1.18,1.24) | < 0.001 |  | 1.31 (1.25,1.37) | < 0.001 |
| WC,(cm) | 1.08 (1.07,1.1) | < 0.001 |  | 1.11 (1.1,1.13) | < 0.001 |
| WHtR(per100) | 1.16 (1.14,1.19) | < 0.001 |  | 1.2 (1.17,1.23) | < 0.001 |
| FM | 1.1 (1.08,1.11) | < 0.001 |  | 1.2 (1.16,1.23) | < 0.001 |
| LM | 1.1 (1.07,1.12) | < 0.001 |  | 1.08 (1.03,1.13) | 0.001 |
| PF | 1.23 (1.2,1.26) | < 0.001 |  | 1.31 (1.26,1.37) | < 0.001 |

ALT, alanine aminotransferase; AST, aspartate aminotransferase; GGT, gamma glutamyl transferase; HDL-c, high‐density lipoprotein cholesterol; TC, total cholesterol; TG, triglyceride; HbA1c, hemoglobin A1c; FPG, fasting plasma glucose; SBP, systolic blood pressure; DBP, diastolic blood pressure; BMI, body mass index; WC, waist circumference; WHtR, waist‐‐height ratio; T2DM, type 2 diabetes mellitus; FM, fat mass; LM, lean mass; PF, per cent fat;

Table S4 Results of univariate and multivariate Cox regression analysis of correlation between BMI, WC, WHtR and T2DM.

|  | Unadjusted | | Adjusted | |
| --- | --- | --- | --- | --- |
| Variable | HR(95%CI) | p value | HR(95%CI) | p value |
| Men |  |  |  |  |
| BMI,(kg/m2) | 1.21 (1.18~1.24) | <0.001 | 1.11 (1.06~1.16) | <0.001 |
| WC,(cm) | 1.08 (1.07~1.1) | <0.001 | 1.04 (1.02~1.06) | <0.001 |
| WHtR100 | 1.16 (1.14~1.19) | <0.001 | 1.08 (1.05~1.11) | <0.001 |
| Women |  |  |  |  |
| BMI,(kg/m2) | 1.31 (1.25~1.37) | <0.001 | 1.15 (1.08~1.23) | <0.001 |
| WC,(cm) | 1.11 (1.1~1.13) | <0.001 | 1.07 (1.04~1.1) | <0.001 |
| WHtR100 | 1.2 (1.17~1.23) | <0.001 | 1.12 (1.07~1.16) | <0.001 |

BMI, body mass index; WC, waist circumference; WHtR, waist‐‐height ratio;

Adjust for age, habit of exercise, smoking status, alcohol consumption, ALT, AST, GGT, HDL, TC, TG, HbA1c, FBP, SBP and DBP levels.


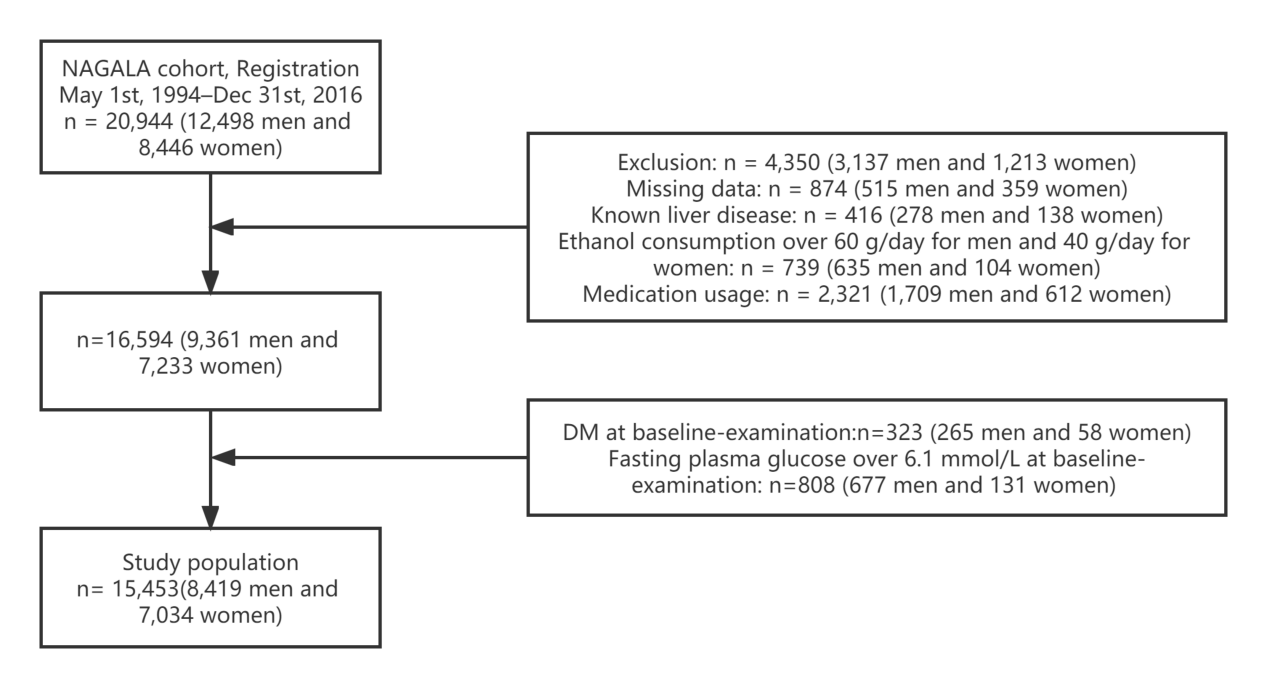


Figure S1 Flowchart of participant selection.


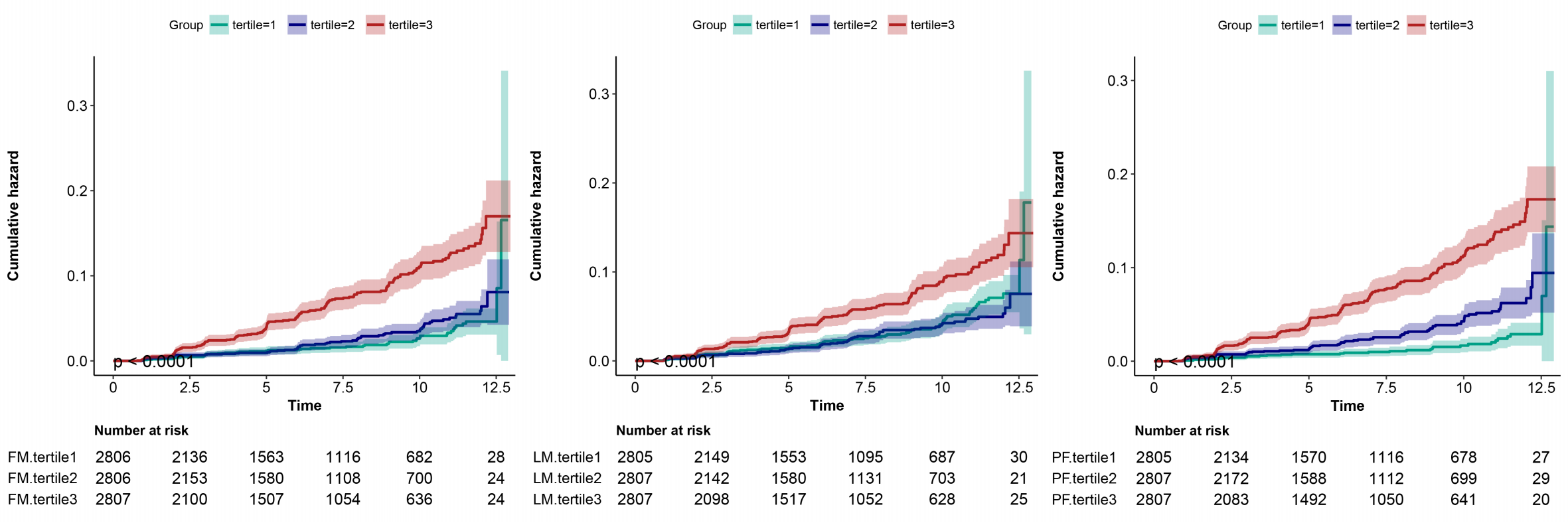


Figure S2a Cumulative incidence of T2DM across tertiles of novel predicted body composition during follow-up in men. The cumulative incidences of T2DM evaluated by Kaplan-Meier analysis were significantly different across the tertiles of predicted FM (A), predicted LM (B) and predicted PF (C) in men participants. People in the tertile 3 had the highest cumulative incidence of T2DM.


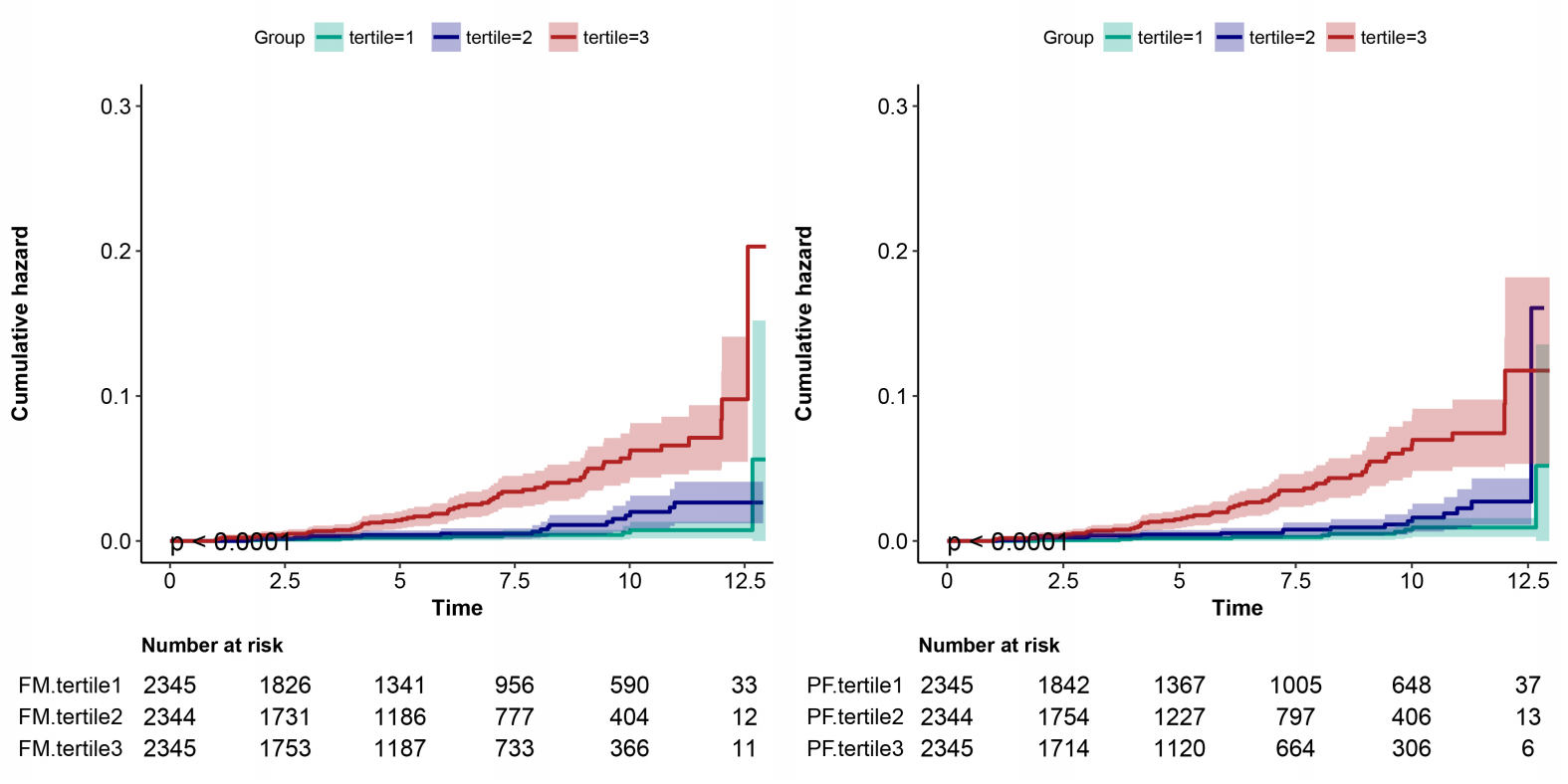


Figure S2b Cumulative incidence of T2DM across tertiles of novel predicted body composition during follow-up in women. The cumulative incidences of T2DM evaluated by Kaplan-Meier analysis were significantly different across the tertiles of predicted FM (A), and predicted PF (B) in women participants. People in tertile 3 had the highest cumulative incidence of T2DM.
